# Synthesis, Structure and Antifungal Activity of New 3-[(5-Aryl-1,3,4-oxadiazol-2-yl)methyl]benzo[*d*]thiazol-2(3*H*)-ones

**DOI:** 10.3390/molecules17010989

**Published:** 2012-01-18

**Authors:** Jian-Quan Weng, Xing-Hai Liu, Hua Huang, Cheng-Xia Tan, Jie Chen

**Affiliations:** 1 College of Chemical Engineering and Materials Science, Zhejiang University of Technology, Hangzhou 310014, China; Email: xhliu@zjut.edu.cn (X.-H.L.); chaohuhuanghua@126.com (H.H.); tanchengxia@zjut.edu.cn (C.-X.T.); 2 Department of Bioassay, Zhejiang Branch of National Southern Pesticide Research Centre, Hangzhou 310023, China; Email: chenjie01@sinochem.com

**Keywords:** 3-[(5-aryl-1,3,4-oxadiazol-2-yl)methyl]benzo[*d*]thiazol-2(3*H*)-ones, synthesis, crystal structure, antifungal activity

## Abstract

A series of new 3-[(5-aryl-1,3,4-oxadiazol-2-yl)methy])benzo[*d*]thiazol-2(3*H*)-ones were synthesized by reaction of (5-substituted-2-oxobenzothiazolin-3-yl)-acetohydrazide with various aromatic acids in POCl_3_ under reflux conditions. The structures of the title compounds were confirmed by ^1^H-NMR, ^13^C-NMR, IR, MS and elemental analysis. Furthermore, the structure of compound **4i** was determined by single-crystal X-ray diffraction. The preliminary bioassy results indicated that some of them showed moderate inhibition activity against *Colletotrichum orbiculare*, *Botrytis cinerea* and *Rhizoctonia solani*.

## 1. Introduction

Benzothiazole derivatives have been found to exhibit broad spectrum of biological effects, such as insecticidal [1], fungicidal [2], antiviral [3], herbicidal [4] and plant-growth-regulating [5] activities, and thus play an important role in research and development of agrochemicals. Meanwhile, 1,3,4-oxadiazole derivatives have gained great importance because of their diverse biological properties, such as insecticidal [6], herbicidal [7] and antifungal [8] activities, and some of them have been successfully commercialized [9,10]. Several synthetic methods for 1,3,4-oxadiazoles were reported. Traditionally, they can be prepared by cyclization of the corresponding acyclic semicarbazide or thiosemicarbazide derivatives using a variety of reagents, including POCl_3_[11], PCl_5_[12], PPA [13], (CF_3_CO)_2_O [14] and so on. Nowadays, new synthetic approaches to 1,3,4-oxadiazoles have been developed, such as microwave assisted synthesis [15], solid phase synthesis [16], Pd-catalyzed [17] and solvent-free [18] methods.

In view of these facts, and as a continuation of our ongoing project aimed at looking for novel biologically active sulfur and nitrogen linked heterocyclic compounds [19,20,21], a series of new 3-[(5-aryl-1,3,4-oxadiazol-2-yl)methyl]benzo[*d*]thiazol-2(3*H*)-ones were synthesized, and their antifungal activities were evaluated.

## 2. Results and Discussion

**Scheme 1:**
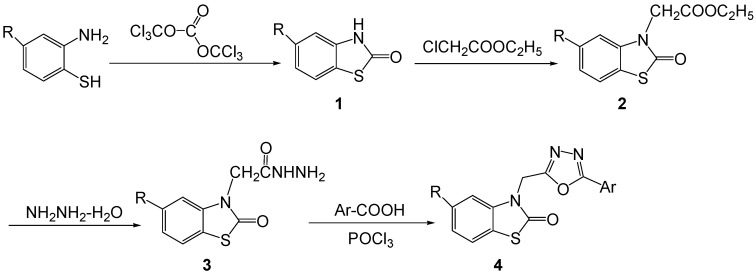
The synthetic route to title compounds **4**.

The synthetic route to the title compounds **4a**–**4v** is shown in Scheme 1.

Substituted 2(3*H*)-benzothiazolones **1** were obtained by the cyclization of 4-substituted*-*2-aminothiophenol with triphosgene, which was seldom reported. The reaction of **1** with ethyl chloroacetate in the presence of potassium carbonate gave the ethyl 2-(2-oxobenzothiazolin-3-yl)acetates **2**. Their acetohyrazides **3** were produced by reaction of compounds **2** and hydrazine hydrate. Finally, the title 3-[(5-aryl-1,3,4-oxadiazol-2-yl)methyl]benzo[*d*]thiazol-2(3*H*)-ones **4a**–**v** were obtained in 69.0–93.2% yields by refluxing acetohydrazides **3** and an aromatic acid in POCl_3_. In general, the yields of compounds **4** bearing chloro in the 5-position are lower than those possessing a hydrogen at that position. Their structures were confirmed by ^1^H-NMR, ^13^C-NMR, IR, MS and elemental analysis. In the ^1^H-NMR spectra of title compounds **4**, the peaks of the 3-position methylene groups appear in the *δ* 5.41–5.51 ppm range, and in the corrsponding ^13^C-NMR spectra, they appear in the *δ* 36.67–37.22 ppm range. In the IR spectra of compounds **4a**–**v**, the characteristic *ν* (C=O) stretching vibration signals appear at 1667–1703 cm^−1^. Meanwhile, all the title compounds exhibited M^+^ or [M+1]^+^ peaks in the MS.

**Figure 1:**
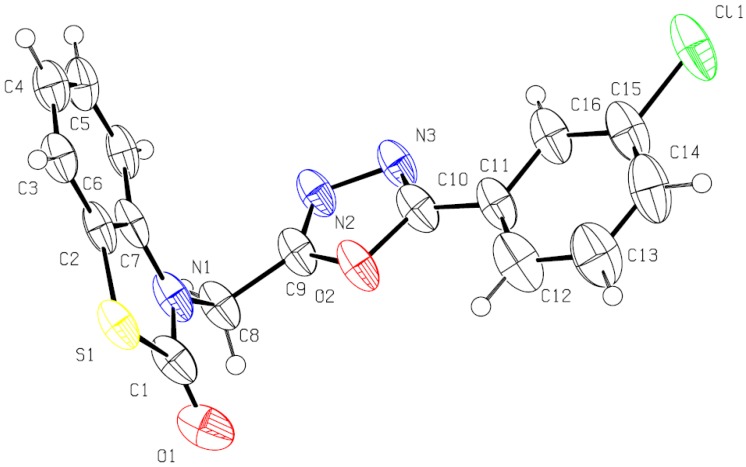
The molecular structure of **4i**.

**Figure 2:**
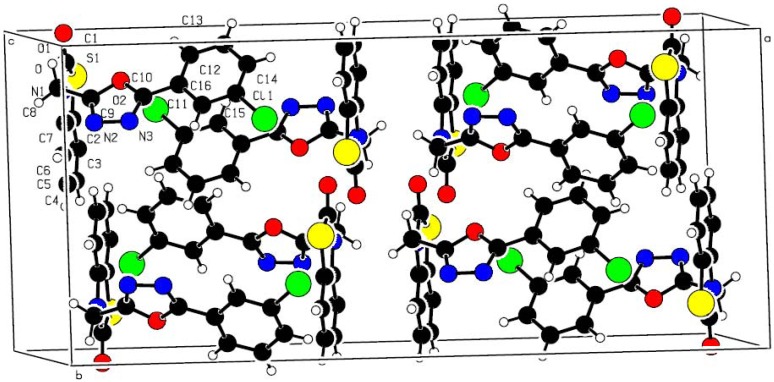
The packing of the molecules in the crystal lattice of **4i**.

When compound **4i** was recrystallized by slow evaporation from acetone, a single crystal was obtained and analyzed by X-ray diffraction crystallography. The molecular structure of compound **4i** is shown in Figure 1 and the packing of the molecule in crystal lattice is illustrated in Figure 2. Its crystal structure is of monoclinic system, space group C2/c with a = 2.718 (2) nm, b = 1.2432 (10) nm, c = 0.9425 (8) nm, α = 90°, β = 108.666 (14)°, γ = 90°, V = 3.017 (4) nm^3^, Z = 8. The bond length of N(2)–N(3) is 0.1420 nm, which is shorter than the normal single N–N bond length (0.1450 nm). The bond lengths are 0.1388 nm and 0.1285 nm for N(1)–C(7) and N(2)–C(9), respectively, which are shorter than the normal single N–C bond length (0.1470 nm) and hence indicative of some double bond character. In the molecular structure of **4i**, the CH_2_ group is nearly perpendicular to the phenyl ring and oxadiazole ring with a θ angle of 110.5°, The oxadiazole ring (O2, C9, N2, N3, C10), phenyl ring (C11, C12, C13, C14, C15, C16), and benzothiazole (C1, S1, C2, C3, C4, C5, C6, C7, N1) are fairly planar with plane equation 4.260x + 8.778y + 5.694z = 4.311, 4.43x + 7.23y + 6.623z = 4.434, 26.998x − 0.047y − 4.027z = −1.253, and the largest deviations from the least squares plane are 0.0037 nm, 0.0054 nm, 0.0189 nm. Meanwhile, the oxadiazole ring is perpendicular to the benzothiazole ring about an angle of 86.1°, and nearly planar with the phenyl ring with an angle of 9.4°.

The *in vitro* antifungal activities of **4a**–**v** against *Colletotrichum orbiculare*, *Botrytis cinerea* and *Rhizoctonia solani* at the dosage of 50 μg/mL were evaluated compared with the commercial fungicide propiconazole. The antifungal activity data are listed in Table 1.

**Table 1:** The inhibition ratios (%) of 4 against *C. orbiculare*, *B. cinerea* and *R. solani* at 50 μg/mL.

Compd.	R	Ar	*C. orbiculare*	*B. cinerea*	*R. solani*
**CK**			0	0	0
**Propiconazole**			87.20	35.00	95.52
**4a**	H	C_6_H_5_-	34.96	50.13	52.57
**4b**	H	2-CH_3_C_6_H_4_-	32.41	14.75	12.02
**4c**	H	3-CH_3_C_6_H_4_-	32.41	16.42	12.64
**4d**	H	4-CH_3_C_6_H_4_-	60.63	44.46	48.19
**4e**	H	4-C_3_H_7_C_6_H_4_-	34.96	48.47	4.88
**4f**	H	4-*i*-C_3_H_7_C_6_H_4_-	28.86	26.77	44.93
**4g**	H	4-*t*-C_4_H_9_C_6_H_4_-	38.50	50.10	42.05
**4h**	H	4-C_5_H_11_C_6_H_4_-	26.31	10.75	2.63
**4i**	H	3-ClC_6_H_4_-	28.86	62.50	30.41
**4j**	H	2,4-Cl_2_C_6_H_3_-	28.82	53.76	4.88
**4k**	H	3-FC_6_H_4_-	34.96	58.81	59.94
**4l**	H	4-FC_6_H_4_-	54.24	72.52	76.37
**4m**	H	2-OCH_3_C_6_H_4_-	38.50	62.50	38.17
**4n**	H	4-OCH_3_C_6_H_4_-	60.34	74.21	48.81
**4o**	H	3-NO_2_C_6_H_4_-	42.05	64.49	48.81
**4p**	H	2-ClC_5_H_3_N-3-	34.96	50.80	42.68
**4q**	Cl	C_6_H_5_-	36.40	48.22	52.02
**4r**	Cl	3-CH_3_C_6_H_4_-	28.64	20.10	13.86
**4s**	Cl	4-CH_3_C_6_H_4_-	61.54	40.50	46.37
**4t**	Cl	4-*t*-C_4_H_9_C_6_H_4_-	41.24	45.60	44.63
**4u**	Cl	4-OCH_3_C_6_H_4_-	64.28	69.50	39.66
**4v**	Cl	2-ClC_5_H_3_N-3-	38.33	52.81	39.02

The preliminary bioassay results showed that all compounds exhibited certain inhibitory activity against all the tested fungi, and some of them possessed moderate antifungal activity. For example, compounds **4d**, **4l**, **4n**, **4s** and **4u** exhibited more than 50% inhibitory activity against *C. orbiculare*, and compounds **4a**, **4k**, **4l** and **4q** displayed greater than 50% inhibition activity against *R. solani*, but their activities were still lower than that of the control fungicide (propiconazole), while most of compounds **4a**–**v** showed activities against *B. cinerea* higher than propiconazole, especially compounds **4i**, **4l**, **4m**, **4n**, **4o** and **4u** showed above 60% inhibition activity against *B. cinerea*. The activity data indicated that compounds **4a**–**v** did not exhibit improved inhibition when a chlorine atom was introduced in 5-position. In general, the compounds bearing an electron-withdrawing group (e.g., halogen and nitro) or methoxy on the aromatic ring (Ar), exhibited higher activity against *B. cinerea* than those bearing an alkyl substituent. It is also note worthy that the inhibition rates of **4a**–**v** against *R. solani* evidently went up when a fluorine atom was introduced on the aromatic ring, especially in the *para*-position (*i.e.*, compound **4l**, which exhibited 76.37% inhibitory activity). Further studies on structural optimization and structure-activity relationships of these title compounds are in progress.

## 3. Experimental

### 3.1. Materials and Reagents

Melting points were determined using an X-4 apparatus without calibration. ^1^H-NMR and ^13^C-NMR spectra were measured on a Bruker ADVANCE III instrument (500 MHz) using TMS as an internal standard and CDCl_3_ or DMSO-*d_6_* as solvents. IR spectra were obtained on a Thermo Nicolet AVATAR 370 FT-IR instrument with KBr plates. Mass spectra were recorded on a Thermo Scientific ITQ 1100TM (EI) or Thermo-Finnigan LCQ-Advantage (ESI) instruments. Elemental analyses were performed on a Vario EL elemental analyzer. X-ray diffraction crystallography was measured on Rigaku Saturn 724 diffractometer. The reaction progress was monitored by TLC plates running in a PE-EtOAc solvent system, and spots were visualized by exposure to UV light (254 nm). All chemical reagents and solvents used in this study were commercial and were used without further purification.

### 3.2. Chemical Synthesis

#### 3.2.1. 5-Substituted-2(3*H*)-benzothiazolones **1a–b**

4-Substituted*-*2-aminothiophenol (0.10 mol) and Et_3_N (33.8 mL, 0.24 mol) were dissolved in CHCl_3_ (30 mL), cooled to 0 °C, and a solution of triphosgene (10.5 g, 0.035 mol) in CHCl_3_ (15 mL) was added dropwise. The resulting mixture was refluxed for 7 h, and then cooled to room temperature, washed with H_2_O (3 × 50 mL), the organic layer was dried over anhydrous MgSO_4_ and evaporated *in vacuo*. The residual solid was recrystallized from ethyl acetate to give pure 5-substituted-2(3*H*)-benzothiazolone (**1**). 

*2(3H)-Benzothiazolone* (**1a**, R = H): yield 71.4%; m.p. 139–141 °C (138–140 °C, lit [22]). ^1^H-NMR (CDCl_3_) δ: 7.14 ~ 7.42 (m, 4H, ArH), 9.75(s, NH, 1H). 

*5-Chloro-2(3H)-benzothiazolone* (**1b**, R = Cl): yield 77.0%; m.p. 240–241 °C (239–240 °C, lit [23]). ^1^H-NMR (CDCl_3_) δ: 7.19 ~ 7.50 (m, 3H, ArH), 9.77 (s, NH, 1H).

#### 3.2.2. Ethyl 2-(5-substituted-2-oxobenzothiazolin-3-yl)acetates **2a–b**

A mixture of 5-substituted-benzo[d]thiazol-2(3*H*)-one **1** (0.1 mol), potassium carbonate (15.9 g, 0.115 mol) and acetone (180 mL) was placed in a flask under stirring. Ethyl chloroacetate (0.11 mol) was added dropwise to the mixture and then refluxed for 6 h. The reaction mixture was next cooled and poured into ice-water. The precipitate formed was filtered and recrystallized from ethanol to give the corresponding ethyl 2-(5-substituted-2-oxobenzothiazolin-3-yl)acetate **2**. 

*Ethyl 2-(2-oxo-benzothiazolin-3-yl)acetate* (**2a**): 20.5 g, yield 92.0%, white solid, m.p. 91–92 °C (90–91 °C, lit [23]). 

*Ethyl 2-(5-chloro-2-oxobenzothiazolin-3-yl)acetate* (**2b**): 24.3 g, yield 94.3%, white solid, m.p. 113–115 °C (115–116 °C, lit [23]).

#### 3.2.3. (5-Substituted-2-oxobenzothiazolin-3-yl)-acetohydrazides **3a–b**

A solution of ethyl (5-substituted-2-oxobenzothiazolin-3-yl)acetate **2** (0.01 mol) and hydrazine hydrate 85% (0.2 mol) in ethanol (100 mL) was refluxed while stirring for 24 h. Then the reaction mixture was cooled, and the precipitate formed was filtered, washed with water and dried to give (5-substituted-2-oxobenzothiazolin-3-yl)-acetohydrazide **3**. 

*(2-Oxobenzothiazolin-3-yl)-acetohydrazide* (**3a**): 1.86 g, yield 83.5%, white solid, m.p. 211–212 °C (211 °C, lit [24]). 

*(5-Chloro-2-oxobenzothiazolin-3-yl)-acetohydrazide* (**3b**): 2.11 g, yield 81.7%, white solid, m.p. 208–210 °C. ^1^H-NMR δ (CDCl_3_): 4.31 (s, 2H, NH_2_), 4.54 (s, 2H, CH_2_), 7.23 ~ 7.68 (m, 3H, ArH), 9.46 (s, 1H, NH).

#### 3.2.4. General Procedure for the Synthesis of Title Compounds **4**

A mixture of (5-substituted-2-oxobenzothiazolin-3-yl)-acetohydrazide **3** (1 mmol), the corresponding aromatic acid (1.05 mmol) and POCl_3_ (5 mL) was heated under refluxing for 8 h. A portion of the POCl_3_ was distilled out and the remaining reaction mixture poured into ice-water. The precipitate formed was filtered and then was purified by column chromatography with PE-EtOAc (V:V = 3:1) to obtain the title 3-((5-aryl-1,3,4-oxadiazol-2-yl)methyl)benzo[*d*]thiazol-2(3*H*)-ones **4a**–**v**.

*3-[(5-Phenyl-1,3,4-oxadiazol-2-yl)methyl]benzo[d]thiazol-2(3H)-one* (**4a**). Yield: 91.4%. Yellow solid, m.p. 166–168 °C; ^1^H-NMR (CDCl_3_) *δ*: 5.47 (s, 2H, CH_2_), 7.21 ~ 7.24 (m, 1H, Ar-H), 7.32 ~ 7.37 (m, 2H, Ar-H), 7.46 ~ 7.51 (m, 3H, Ar-H), 7.55 (t, *J* = 7.5 Hz, 1H, Ar-H), 8.02 (d, *J* = 7.5 Hz, 2H, Ar-H); ^13^C-NMR (DMSO-*d_6_*) *δ*: 168.95, 164.66, 161.53, 136.20, 132.16, 129.43, 126.76, 126.51, 123.69, 123.07, 122.95, 121.20, 111.59, 36.94; IR (cm^−1^) *ν*: 3057, 2988, 1682, 1592, 1475, 1324, 1186, 1016, 774, 747, 710, 687; EI-MS *m/z* (relative intensity): 310 (M+1, 16), 309 (M^+^, 82), 281 (16), 159 (14), 136 (100), 109 (24), 105 (81), 77 (53); Elemental anal. (%), calcd. for C_16_H_11_N_3_O_2_S: C, 62.12; H, 3.58; N, 13.58; found: C, 62.31; H, 3.55; N, 13.62.

*3-[(5-o-Tolyl-1,3,4-oxadiazol-2-yl)methyl]benzo[d]thiazol-2(3H)-one* (**4b**). Yield: 89.7%. Yellow solid, m.p. 187–189 °C; ^1^H-NMR (CDCl_3_) *δ*: 2.65 (s, 3H, CH_3_), 5.48 (s, 2H, CH_2_), 7.20 ~ 7.24 (m, 1H, Ar-H), 7.29 ~ 7.37 (m, 4H, Ar-H), 7.42 (t, *J* = 7.0 Hz, 1H, Ar-H), 7.46 (d, *J* = 7.5 Hz, 1H, Ar-H), 7.89 (d, *J* = 7.5 Hz, 1H, Ar-H); ^13^C-NMR (DMSO-*d_6_*) *δ*: 168.95, 164.84, 161.09, 137.57, 136.20, 131.72, 131.61, 128.69, 126.75, 126.47, 123.70, 123.07, 122.13, 121.20, 111.62, 36.88, 21.14; IR (cm^−1^) *ν*: 3071, 2973, 1674, 1592, 1475, 1390, 1331, 1184, 1133, 1070, 1020, 745; EI-MS *m/z* (relative intensity): 324 (M+1, 7), 323 (M^+^, 34), 136 (100), 119 (49), 109 (17), 91 (34), 77 (10), 65 (13); Elemental anal. (%), calcd. for C_17_H_13_N_3_O_2_S: C, 63.14; H, 4.05; N, 12.99; found: C, 63.33; H, 4.03; N, 13.04.

*3-[(5-m-Tolyl-1,3,4-oxadiazol-2-yl)methyl]benzo[d]thiazol-2(3H)-one* (**4c**). Yield: 87.4%. Yellow solid, m.p. 146–148 °C; ^1^H-NMR (CDCl_3_) *δ*: 2.65 (s, 3H, CH_3_), 5.48 (s, 2H, CH_2_), 7.20 ~ 7.23 (m, 1H, Ar-H), 7.34 ~ 7.39 (m, 4H, Ar-H), 7.46 (d, *J* = 7.5 Hz, 1H, Ar-H), 7.81 (d, *J* = 7.5 Hz, 1H, Ar-H), 7.85 (s, 1H, Ar-H); ^13^C-NMR (DMSO-*d_6_*) *δ*: 168.94, 164.74, 161.45, 138.93, 136.19, 132.82, 129.33, 126.78, 126.74, 123.71, 123.68, 123.06, 122.87, 121.19, 111.58, 36.93, 20.74; IR (cm^−1^) *ν*: 3074, 2954, 1682, 1589, 1549, 1473, 1323, 1183, 1081, 763, 726, 583; EI-MS *m/z* (relative intensity): 324 (M+1, 21), 323 (M^+^, 100), 173 (16), 136 (84), 119 (88), 109 (19), 91 (50), 77 (11); Elemental anal. (%), calcd. for C_17_H_13_N_3_O_2_S: C, 63.14; H, 4.05; N, 12.99; found: C, 63.37; H, 4.03; N, 13.03.

*3-[(5-p-Tolyl-1,3,4-oxadiazol-2-yl)methyl]benzo[d]thiazol-2(3H)-one* (**4d**). Yield: 90.2%. Yellow solid, m.p. 183–185 °C; ^1^H-NMR (CDCl_3_) *δ*: 2.42 (s, 3H, CH_3_), 5.45 (s, 2H, CH_2_), 7.20 ~ 7.23 (m, 1H, Ar-H), 7.29 (d, *J* = 8.0 Hz, 2H, Ar-H), 7.33 ~ 7.35 (m, 2H, Ar-H), 7.50 (d, *J* = 7.5 Hz, 1H, Ar-H), 7.90 (d, *J* = 8.0 Hz, 2H, Ar-H); ^13^C-NMR (CDCl_3_) *δ*: 169.88, 166.12, 160.35, 142.80, 135.93, 129.76, 127.08, 126.87, 123.99, 122.80, 122.24, 120.40, 111.03, 36.74, 21.64; IR (cm^−1^) *ν*: 3078, 2920, 1685, 1614, 1591, 1499, 1474, 1323, 1181, 1068, 745, 470; EI-MS *m/z* (relative intensity): 324 (M+1, 11), 323 (M^+^, 52), 173 (19), 136 (73), 119 (100), 109 (15), 91 (37), 65 (15); Elemental anal. (%), calcd. for C_17_H_13_N_3_O_2_S: C, 63.14; H, 4.05; N, 12.99; found: C, 63.29; H, 4.03; N, 12.95.

*3-[(5-(4-Propylphenyl)-1,3,4-oxadiazol-2-yl)methyl]benzo[d]thiazol-2(3H)-one* (**4e**). Yield: 86.0%. Pale solid, m.p. 145–147 °C; ^1^H-NMR (CDCl_3_) *δ*: 0.95 (t, *J* = 7.5Hz, 3H, CH_3_), 1.65 ~ 1.69 (m, 2H, CH_2_CH_2_CH_3_), 2.65 (t, *J* = 7.5 Hz, 2H, CH_2_CH_2_CH_3_), 5.45 (s, 2H, CH_2_), 7.21 ~ 7.23 (m, 1H, Ar-H), 7.29 (d, *J* = 8.5 Hz, 2H, Ar-H), 7.32 ~ 7.34 (m, 2H, Ar-H), 7.45 (d, *J* = 8.0 Hz, 1H, Ar-H), 7.92 (d, *J* = 8.0 Hz, 2H, Ar-H); ^13^C-NMR (DMSO-*d_6_*) *δ*: 168.93, 164.75, 161.27, 146.37, 136.20, 129.38, 128.34, 127.43, 123.69, 123.25, 121.20, 120.47, 111.58, 43.20, 36.97, 23.70, 13.49; IR (cm^−1^) *ν*: 3032, 2960, 1680, 1605, 1566, 1474, 1409, 1360, 1332, 1243, 1185, 755; EI-MS *m/z* (relative intensity): 352 (M+1, 6), 351 (M^+^, 27), 201 (15), 147 (100), 136 (93), 116 (19), 109 (14), 91 (19); Elemental anal. (%), calcd. for C_19_H_17_N_3_O_2_S: C, 64.94; H, 4.88; N, 11.96; found: C, 65.06; H, 4.85; N, 12.01.

*3-[(5-(4-Isopropylphenyl)-1,3,4-oxadiazol-2-yl)methyl]benzo[d]thiazol-2(3H)-one* (**4f**). Yield: 83.8%. Yellow solid, m.p. 143–145 °C; ^1^H-NMR (CDCl_3_) *δ*: 1.27 (d, *J* = 7.0 Hz, 6H, CH(CH_3_)_2_), 2.94 ~ 2.99 (m, 1H, CH(CH_3_)_2_), 5.46 (s, 2H, CH_2_), 7.20 ~ 7.23 (m, 1H, Ar-H), 7.33 ~ 7.35 (m, 4H, Ar-H), 7.47 (d, *J* = 6.0 Hz, 1H, Ar-H), 7.93 (d, *J* = 8.0 Hz, 2H, Ar-H); ^13^C-NMR (DMSO-*d_6_*) *δ*: 168.93, 164.70, 161.28, 152.92, 136.20, 127.40, 126.74, 126.63, 123.68, 123.24, 121.18, 120.57, 111.58, 36.92, 33.40, 23.40; IR (cm^−1^) *ν*: 3071, 2964, 1687, 1614, 1593, 1476, 1421, 1322, 1181, 1012, 842, 741; EI-MS *m/z* (relative intensity): 352 (M+1, 14), 351 (M^+^, 59), 147 (78), 136 (100), 130 (16), 109 (13), 103 (11), 91 (13); Elemental anal. (%), calcd. for C_19_H_17_N_3_O_2_S: C, 64.94; H, 4.88; N, 11.96; found: C, 65.09; H, 4.86; N, 12.04.

*3-[(5-(4-tert-Butylphenyl)-1,3,4-oxadiazol-2-yl)methyl]benzo[d]thiazol-2(3H)-one* (**4g**). Yield: 78.5%. Yellow solid, m.p. 188–190 °C; ^1^H-NMR (CDCl_3_) *δ*: 1.35 (s, 9H, C(CH_3_)_3_), 5.46 (s, 2H, CH_2_), 7.19 ~ 7.23 (m, 1H, Ar-H), 7.31 ~ 7.35 (m, 2H, Ar-H), 7.45 (d, *J* = 7.5 Hz, 1H, Ar-H), 7.49 (d, *J* = 8.5 Hz, 2H, Ar-H), 7.93 (d, *J* = 8.0 Hz, 2H, Ar-H); ^13^C-NMR (CDCl_3_) *δ*: 169.86, 166.05, 160.37, 155.86, 135.92, 126.96, 126.86, 126.04, 123.98, 122.80, 122.23, 120.34, 111.00, 36.76, 35.09, 31.05; IR (cm^−1^) *ν*: 3061, 2968, 1685, 1615, 1593, 1477, 1322, 1180, 1116, 1011, 844, 742; EI-MS *m/z* (relative intensity): 366 (M+1, 7), 365 (M^+^, 32), 350 (8), 161 (66), 144 (11), 136 (100), 116 (13), 91 (8); Elemental anal. (%), calcd. for C_20_H_19_N_3_O_2_S: C, 65.73; H, 5.24; N, 11.50; found: C, 65.81; H, 5.22; N, 11.54.

*3-[(5-(4-Pentylphenyl)-1,3,4-oxadiazol-2-yl)methyl]benzo[d]thiazol-2(3H)-one* (**4h**). Yield: 81.1%. Pale solid, m.p. 196–198 °C; ^1^H-NMR (CDCl_3_) *δ*: 0.90(t, *J* = 7.0 Hz, 3H, CH_3_), 1.32 ~ 1.65 (m, 6H, CH_2_(CH_2_)_3_CH_3_), 2.66 (t, *J* = 7.5 Hz, 2H, CH_2_(CH_2_)_3_CH_3_), 5.46 (s, 2H, CH_2_), 7.19 ~ 7.23 (m, 1H, Ar-H), 7.29 (d, *J* = 8.5 Hz, 2H, Ar-H), 7.38 (t, *J* = 7.5 Hz, 1H, Ar-H), 7.47 (t, *J* = 7.5 Hz, 2H, Ar-H), 7.91 (d, *J* = 8.5 Hz, 2H, Ar-H); ^13^C-NMR (DMSO-*d_6_*) *δ*: 168.99, 164.81, 161.29, 147.15, 136.23, 129.35, 126.81, 126.56, 123.74, 123.10, 121.24, 120.45, 111.62, 36.95, 34.99, 30.78, 30.19, 21.88, 13.85; IR (cm^−1^) *ν*: 3031, 2929, 1667, 1612, 1595, 1476, 1340, 1242, 1190, 1023, 853, 748; EI-MS *m/z* (relative intensity): 380 (M+1, 23), 379 (M^+^, 97), 229 (13), 175 (92), 136 (100), 118 (13), 109 (13), 91 (22); Elemental anal. (%), calcd. for C_21_H_21_N_3_O_2_S: C, 66.47; H, 5.58; N, 11.07; found: C, 66.54; H, 5.55; N, 11.11.

*3-[(5-(3-Chlorophenyl)-1,3,4-oxadiazol-2-yl)methyl]benzo[d]thiazol-2(3H)-one* (**4i**). Yield: 85.8%. White solid, m.p. 171–173 °C; ^1^H-NMR (CDCl_3_) *δ*: 5.47 (s, 2H, CH_2_), 7.20 ~ 7.24 (m, 1H, Ar-H), 7.31 ~ 7.36 (m, 2H, Ar-H), 7.44 ~ 7.48 (m, 2H, Ar-H), 7.51 ~ 7.54 (m, 1H, Ar-H), 7.91 (d, *J* = 7.5 Hz, 1H, Ar-H), 8.02 (s, 1H, Ar-H); ^13^C-NMR (CDCl_3_) *δ*: 169.89, 164.84, 160.98, 135.82, 135.27, 132.23, 130.44, 127.07, 126.91, 125.22, 124.79, 124.09, 122.89, 122.26, 110.91, 36.67; IR (cm^−1^) *ν*: 3064, 2960, 1699, 1594, 1571, 1477, 1424, 1325, 1242, 1180, 801, 746, 585; EI-MS *m/z* (relative intensity): 345 (M+2, 31), 344 (M+1, 17), 343 (M^+^, 74), 314 (16), 193 (12), 141 (28), 136 (100), 111 (41), 75 (16); Elemental anal. (%), calcd. for C_16_H_10_ClN_3_O_2_S: C, 55.90; H, 2.93; N, 12.22; found: C, 56.07; H, 2.91; N, 12.27.

*3-[(5-(2,4-Dichlorophenyl)-1,3,4-oxadiazol-2-yl)methyl]benzo[d]thiazol-2(3H)-one* (**4j**). Yield: 84.6%. White solid, m.p. 162–163 °C; ^1^H-NMR (CDCl_3_) *δ*: 5.49 (s, 2H, CH_2_), 7.21 ~ 7.25 (m, 1H, Ar-H), 7.29 (d, *J* = 8.0 Hz, 1H, Ar-H), 7.33 ~ 7.36 (m, 1H, Ar-H), 7.38 ~ 7.40 (m, 1H, Ar-H), 7.47 (d, *J* = 7.5 Hz, 1H, Ar-H), 7.56 (d, *J* = 2.0 Hz, 1H, Ar-H), 7.91 (d, *J* = 8.5 Hz, 1H, Ar-H); ^13^C-NMR (CDCl_3_) *δ*: 169.85, 163.55, 161.15, 138.58, 135.79, 134.14, 131.93, 131.25, 127.64, 126.89, 124.08, 122.89, 122.29, 120.99, 110.89, 36.69; IR (cm^−1^) *ν*: 3095, 2922, 1674, 1593, 1474, 1418, 1375, 1334, 1184, 1104, 1023, 816, 746; EI-MS *m/z* (relative intensity): 382 (M+4, 1), 380 (M+2, 5), 379 (M+1, 22), 378 (M^+^, 8), 377 (M−1, 45), 173 (61), 164 (16), 145 (14), 136 (100), 109 (31); Elemental anal. (%), calcd. for C_16_H_9_Cl_2_N_3_O_2_S: C, 50.81; H, 2.40; N, 11.11; found: C, 50.97; H, 2.38; N, 11.15.

*3-[(5-(3-Fluorophenyl)-1,3,4-oxadiazol-2-yl)methyl]benzo[d]thiazol-2(3H)-one* (**4k**). Yield: 87.5%. Yellow solid, m.p. 174–176 °C; ^1^H-NMR (CDCl_3_) *δ*: 5.47 (s, 2H, CH_2_), 7.18 ~ 7.24 (m, 2H, Ar-H), 7.28 ~ 7.34 (m, 2H, Ar-H), 7.44 ~ 7.48 (m, 2H, Ar-H), 7.69 ~ 7.71 (m, 1H, Ar-H), 7.79 (d, *J* = 7.5 Hz, 1H, Ar-H); ^13^C-NMR (CDCl_3_) *δ*: 169.85, 164.98, 164.95, 163.75, 161.78, 160.93, 135.83, 130.99, 130.92, 126.90, 125.07, 125.00, 124.07, 122.92, 122.90, 122.88, 122.27, 119.35, 119.18, 114.27, 114.08, 110.90, 36.68; IR (cm^−1^) *ν*: 3082, 2951, 1681, 1591, 1473, 1423, 1323, 1270, 1194, 1184, 869, 766, 728; EI-MS *m/z* (relative intensity): 328 (M+1, 19), 327 (M^+^, 100), 299 (20), 177 (13), 136 (98), 123 (98), 109 (27), 95 (65); Elemental anal. (%), calcd. for C_16_H_10_FN_3_O_2_S: C, 58.71; H, 3.08; N, 12.84; found: C, 58.85; H, 3.06; N, 12.80.

*3-[(5-(4-Fluorophenyl)-1,3,4-oxadiazol-2-yl)methyl]benzo[d]thiazol-2(3H)-one* (**4l**). Yield: 82.6%. Yellow solid, m.p. 192–194 °C; ^1^H-NMR (CDCl_3_) *δ*: 5.46 (s, 2H, CH_2_), 7.17 ~ 7.24 (m, 3H, Ar-H), 7.32 ~ 7.37 (m, 2H, Ar-H), 7.46 ~ 7.48 (m, 1H, Ar-H), 8.02 ~ 8.06 (m, 2H, Ar-H); ^13^C-NMR (CDCl_3_) *δ*: 169.86, 166.03, 165.16, 164.01, 160.64, 135.87, 129.52, 129.45, 126.89, 124.05, 122.85, 122.25, 119.54, 119.52, 116.55, 116.37, 110.96, 36.68; IR (cm^−1^) *ν*: 3075, 3006, 1681, 1607, 1495, 1472, 1323, 1235, 1176, 1156, 1087, 848, 750; EI-MS *m/z* (relative intensity): 328 (M+1, 16), 327 (M^+^, 85), 299 (16), 177 (20), 136 (77), 123 (100), 109 (24), 95 (43); Elemental anal. (%), calcd. for C_16_H_10_FN_3_O_2_S: C, 58.71; H, 3.08; N, 12.84; found: C, 58.82; H, 3.07; N, 12.88.

*3-[(5-(2-Methoxyphenyl)-1,3,4-oxadiazol-2-yl)methyl]benzo[d]thiazol-2(3H)-one* (**4m**). Yield: 91.5%. Yellow solid, m.p. 173–174 °C; ^1^H-NMR (CDCl_3_) *δ*: 3.92 (s, 3H, OCH_3_), 5.47 (s, 2H, CH_2_), 7.02 ~ 7.07 (m, 2H, Ar-H), 7.20 ~ 7.23 (m, 1H, Ar-H), 7.33 ~ 7.34 (m, 2H, Ar-H), 7.45 (d, *J* = 7.5 Hz, 1H, Ar-H), 7.49 ~ 7.52 (m, 1H, Ar-H), 7.87 ~ 7.89 (dd, *J*_1_ = 2.0 Hz, *J*_2_ = 7.5 Hz, 1H, Ar-H); ^13^C-NMR (CDCl_3_) *δ*: 169.81, 164.65, 160.29, 157.97, 135.99, 133.45, 130.54, 126.81, 123.91, 122.73, 122.24, 120.72, 112.35, 111.96, 111.09, 55.93, 36.80; IR (cm^−1^) *ν*: 3090, 2995, 1686, 1605, 1592, 1496, 1474, 1323, 1269, 1181, 1022, 751; EI-MS *m/z* (relative intensity): 340 (M+1, 12), 339 (M^+^, 56), 237 (14), 175 (20), 164 (25), 136 (100), 109 (26), 77 (22); Elemental anal. (%), calcd. for C_17_H_13_N_3_O_3_S: C, 60.17; H, 3.86; N, 12.38; found: C, 60.33; H, 3.88; N, 12.46.

*3-[(5-(4-Methoxyphenyl)-1,3,4-oxadiazol-2-yl)methyl]benzo[d]thiazol-2(3H)-one* (**4n**). Yield: 93.2%. Yellow solid, m.p. 142–143 °C; ^1^H-NMR (CDCl_3_) *δ*: 3.88 (s, 3H, OCH_3_), 5.44 (s, 2H, CH_2_), 6.98 ~ 7.00 (m, 2H, Ar-H), 7.21 ~ 7.23 (m, 1H, Ar-H), 7.34 ~ 7.35 (m, 2H, Ar-H), 7.46 (d, *J* = 7.5 Hz, 1H, Ar-H), 7.95 ~ 7.97 (m, 2H, Ar-H); ^13^C-NMR (DMSO-*d_6_*) *δ*: 168.94, 164.59, 162.14, 160.94, 136.22, 128.38, 126.76, 123.69, 123.07, 121.20, 115.26, 114.90, 111.60, 55.49, 36.92; IR (cm^−1^) *ν*: 3086, 2979, 1671, 1593, 1499, 1474, 1325, 1309, 1257, 1179, 1020, 842, 762; EI-MS *m/z* (relative intensity): 340 (M+1, 5), 339 (M^+^, 24), 189 (15), 135 (100), 133 (24), 109 (10), 92 (8), 77 (10); Elemental anal. (%), calcd. for C_17_H_13_N_3_O_3_S: C, 60.17; H, 3.86; N, 12.38; found: C, 60.38; H, 3.82; N, 12.44.

*3-[(5-(3-Nitrophenyl)-1,3,4-oxadiazol-2-yl)methyl]benzo[d]thiazol-2(3H)-one* (**4o**). Yield: 82.6%. Yellow solid, m.p. 158–160 °C; ^1^H-NMR (CDCl_3_) *δ*: 5.51 (s, 2H, CH_2_), 7.22 ~ 7.24 (m, 1H, Ar-H), 7.30 ~ 7.33 (m, 1H, Ar-H), 7.35 ~ 7.39 (m, 1H, Ar-H), 7.48 (d, *J* = 8.0 Hz, 1H, Ar-H), 7.74 (t, *J* = 8.0 Hz, 1H, Ar-H), 8.37 ~ 8.39 (m, 1H, Ar-H), 8.41 ~ 8.43 (m, 1H, Ar-H), 8.87 ~ 8.88 (m, 1H, Ar-H); ^13^C-NMR (DMSO-*d_6_*) *δ*: 168.99, 163.24, 162.19, 148.18, 136.17, 132.61, 131.40, 126.62, 126.52, 124.46, 123.73, 123.09, 121.22, 121.10, 111.63, 36.92; IR (cm^−1^) *ν*: 3085, 2926, 1689, 1595, 1529, 1474, 1426, 1351, 1327, 1230, 1183, 744, 713; EI-MS *m/z* (relative intensity): 355 (M+1, 4), 354 (M^+^, 15), 322 (100), 265 (85), 150 (27), 136 (18), 91 (50), 77 (14); Elemental anal. (%), calcd. for C_16_H_10_N_4_O_4_S: C, 54.23; H, 2.84; N, 15.81; found: C, 54.40; H, 2.82; N, 15.87.

*3-[(5-(2-Chloropyridin-3-yl)-1,3,4-oxadiazol-2-yl)methyl]benzo[d]thiazol-2(3H)-one* (**4p**). Yield: 73.5%. Yellow solid, m.p. 169–171 °C; ^1^H-NMR (CDCl_3_) *δ*: 5.51 (s, 2H, CH_2_), 7.24 (t, *J* = 7.5 Hz, 1H, Ar-H), 7.29 (d, *J* = 8.0 Hz, 1H, Ar-H), 7.37 (t, *J* = 7.5 Hz, 1H, Ar-H), 7.41 ~ 7.44 (m, 1H, Ar-H), 7.48 ~ 7.49 (m, 1H, Ar-H), 8.31 ~ 8.33 (m, 1H, Ar-H), 8.58 ~ 8.59 (m, 1H, Ar-H); ^13^C-NMR (DMSO-*d_6_*) *δ*: 168.98, 162.37, 161.68, 152.50, 147.72, 140.56, 136.15, 126.77, 123.75, 123.54, 123.10, 121.22, 119.51, 111.66, 36.87; IR (cm^−1^) *ν*: 3089, 2923, 1703, 1593, 1542, 1475, 1427, 1380, 1323, 1178, 1061, 748; EI-MS *m/z* (relative intensity): 346 (M+2, 15), 344 (M^+^, 55), 164 (11), 142 (12), 136 (100), 112 (22), 109 (27), 76 (15); Elemental anal. (%), calcd. for C_1__5_H_9_ClN_4_O_2_S: C, 52.25; H, 2.63; N, 16.25; found: C, 52.47; H, 2.62; N, 16.31.

*5-Chloro-3-[(5-phenyl-1,3,4-oxadiazol-2-yl)methyl)]benzo[d]thiazol-2(3H)-one* (**4q**). Yield: 83.6%. Yellow solid, m.p. 218–220 °C; ^1^H-NMR (CDCl_3_) δ: 5.43 (s, 2H, CH_2_), 7.20 ~ 7.22 (m, 1H, Ar-H), 7.36 ~ 7.39 (m, 2H, Ar-H), 7.50 ~ 7.53 (m, 2H, Ar-H), 7.55 ~ 7.59 (m, 1H, Ar-H), 8.03 (d, *J* = 7.0 Hz, 2H, Ar-H); ^13^C-NMR (DMSO-*d_6_*) *δ*: 169.15, 164.74, 161.36, 137.46, 132.20, 131.49, 129.48, 126.54, 124.56, 123.52, 123.01, 120.08, 111.90, 37.22; IR (cm^−1^) *ν*: 3097, 2950, 1692, 1591, 1473, 1440, 1322, 1240, 1169, 1089, 800, 712; ESI-MS *m/z*: 344 [M+H]^+^, 345 [M+2]^+^, 366 [M+Na]^+^. Elemental anal. (%), calcd. for C_16_H_10_ClN_3_O_2_S: C, 55.90; H, 2.93; N, 12.22; found: C, 56.16; H, 2.91; N, 12.24.

*5-Chloro-3-[(5-m-tolyl-1,3,4-oxadiazol-2-yl)methyl]benzo[d]thiazol-2(3H)-one* (**4r**). Yield: 81.5%. Yellow solid, m.p. 209–211 °C; ^1^H-NMR (CDCl_3_) *δ*: 2.43 (s, 3H, CH_3_), 5.43 (s, 2H, CH_2_), 7.20 ~ 7.22 (m, 1H, Ar-H), 7.36 ~ 7.38 (m, 3H, Ar-H), 7.39 (s, 1H, Ar-H), 7.82 (d, *J* = 7.0 Hz, 1H, Ar-H), 7.86 (s, 1H, Ar-H); ^13^C-NMR (DMSO-*d_6_*) *δ*: 169.15, 164.81, 161.28, 138.97, 137.46, 132.86, 131.49, 129.38, 126.83, 124.56, 123.73, 123.52, 122.94, 120.09, 111.91, 37.20, 20.78; IR (cm^−1^) *ν*: 3091, 2951, 1682, 1591, 1473, 1441, 1324, 1182, 1140, 1087, 893, 799; ESI-MS *m/z*: 358 [M+H]^+^, 359 [M+2]^+^, 380 [M+Na]^+^. Elemental anal. (%), calcd. for C_1__7_H_1__2_ClN_3_O_2_S: C, 57.06; H, 3.38; N, 11.74; found: C, 57.32; H, 3.36; N, 11.77.

*5-Chloro-3-[(5-p-tolyl-1,3,4-oxadiazol-2-yl)methyl]benzo[d]thiazol-2(3H)-one* (**4s**). Yield: 83.0%. Yellow solid, m.p. 109–110 °C; ^1^H-NMR (CDCl_3_) *δ*: 2.43 (s, 3H, CH_3_), 5.42 (s, 2H, CH_2_), 7.20 ~ 7.22 (m, 1H, Ar-H), 7.30 (d, *J* = 8.0 Hz, 2H, Ar-H), 7.36 (d, *J* = 2.0 Hz, 1H, Ar-H), 7.37 (d, *J* = 8.5 Hz, 1H, Ar-H), 7.91 (d, *J* = 8.0 Hz, 2H, Ar-H); ^13^C-NMR (DMSO-*d_6_*) *δ*: 169.12, 164.81, 161.06, 142.37, 137.44, 131.48, 129.98, 126.47, 124.52, 123.49, 120.25, 120.07, 111.87, 37.19, 21.08; IR (cm^−1^) *ν*: 3094, 2923, 1686, 1591, 1490, 1471, 1440, 1317, 1182, 1087, 825, 730; ESI-MS *m/z*: 358 [M+H]^+^, 359 [M+2]^+^, 380 [M+Na]^+^. Elemental anal. (%), calcd. for C_1__7_H_1__2_ClN_3_O_2_S: C, 57.06; H, 3.38; N, 11.74; found: C, 57.14; H, 3.39; N, 11.78.

*3-[(5-(4-tert-Butylphenyl)-1,3,4-oxadiazol-2-yl)methyl]-5-chlorobenzo[d]thiazol-2(3H)-one* (**4t**). Yield: 73.0%. Yellow solid, m.p. 180–181 °C; ^1^H-NMR (CDCl_3_) *δ*: 1.35 (s, 9H, C(CH_3_)_3_), 5.42 (s, 2H, CH_2_), 7.19 ~ 7.21 (m, 1H, Ar-H), 7.35 (d, *J* = 2.0 Hz, 1H, Ar-H), 7.37 (d, *J* = 8.5 Hz, 1H, Ar-H), 7.51 (d, *J* = 8.5 Hz, 2H, Ar-H), 7.95 (d, *J* = 8.5 Hz, 2H, Ar-H); ^13^C-NMR (DMSO-*d_6_*) *δ*: 169.12, 164.72, 161.13, 155.15, 137.45, 131.48, 126.38, 126.28, 124.54, 123.50, 120.27, 120.07, 111.87, 37.20, 34.80, 30.74; IR (cm^−1^) *ν*: 3096, 2921, 1681, 1591, 1494, 1475, 1447, 1337, 1189, 1113, 852, 811; ESI-MS *m/z*: 400 [M+H]^+^, 401 [M+2]^+^, 422 [M+Na]^+^. Elemental anal. (%), calcd. for C_20_H_1__8_ClN_3_O_2_S: C, 60.07; H, 4.54; N, 10.51; found: C, 60.31; H, 4.51; N, 10.55.

*5-Chloro-3-[(5-(4-methoxyphenyl)-1,3,4-oxadiazol-2-yl)methyl]benzo[d]thiazol-2(3H)-one* (**4u**). Yield: 85.8%. Yellow solid, m.p. 170–171 °C; ^1^H-NMR (CDCl_3_) *δ*: 3.88 (s, 3H, OCH_3_), 5.41 (s, 2H, CH_2_), 6.99 ~ 7.01 (m, 2H, Ar-H), 7.19 ~ 7.21 (m, 1H, Ar-H), 7.36 ~ 7.39 (m, 2H, Ar-H), 7.96 (d, *J* = 9.0 Hz, 2H, Ar-H); ^13^C-NMR (DMSO-*d_6_*) *δ*: 169.12, 164.65, 162.15, 160.76, 137.46, 131.48, 128.38, 124.54, 123.49, 120.07, 115.30, 114.92, 111.87, 55.50, 37.18; IR (cm^−1^) *ν*: 3094, 2922, 1687, 1593, 1501, 1475, 1326, 1259, 1180, 1088, 1026, 837, 800; ESI-MS *m/z*: 374 [M+H]^+^, 375 [M+2]^+^, 396 [M+Na]^+^. Elemental anal. (%), calcd. for C_17_H_1__2_ClN_3_O_3_S: C, 54.62; H, 3.24; N, 11.24; found: C, 54.77; H, 3.27; N, 11.26.

*5-Chloro-3-[(5-(2-chloropyridin-3-yl)-1,3,4-oxadiazol-2-yl)methyl]benzo[d]thiazol-2(3H)-one* (**4v**). Yield: 69.0%. Yellow solid, m.p. 165–167 °C; ^1^H-NMR (CDCl_3_) *δ*: 5.48 (s, 2H, CH_2_), 7.22 ~ 7.24 (dd, *J*_1_ = 2.0 Hz, *J*_2_ = 8.5 Hz, 1H, Ar-H), 7.32 (d, *J* = 2.0 Hz, 1H, Ar-H), 7.39 (d, *J* = 8.5 Hz, 1H, Ar-H), 7.42 ~ 7.45 (m, 1H, Ar-H), 8.35 ~ 8.37 (dd, *J*_1_ = 2.0 Hz, *J*_2_ = 7.5 Hz, 1H, Ar-H), 8.59 ~ 8.60 (dd, *J*_1_ = 2.0 Hz, *J*_2_ = 4.5 Hz, 1H, Ar-H); ^13^C-NMR (DMSO-*d_6_*) *δ*: 169.13, 162.17, 161.73, 152.51, 147.67, 140.54, 137.36, 131.48, 124.55, 123.54, 120.07, 119.50, 111.96, 37.06; IR (cm^−1^) *ν*: 3077, 2976, 1693, 1591, 1575, 1473, 1441, 1391, 1340, 1184, 809, 742; ESI-MS *m/z*: 379 [M+H]^+^, 380 [M+2]^+^, 382 [M+4]^+^, 401 [M+Na]^+^. Elemental anal. (%), calcd. for C_15_H_8_Cl_2_N_4_O_2_S: C, 47.51; H, 2.13; N, 14.77; found: C, 47.70; H, 2.12; N, 14.82.

### 3.3. Crystal Structure Determination

The crystal of compound **4i** with dimensions of 0.20 mm × 0.18 mm × 0.16 mm was mounted on a Rigaku Saturn 724 diffractometer with a graphite-monochromated Mo*Kα* radiation (λ = 0.71073 Å) by using a Phi scan modes at 113 (2) K in the reange of 1.82° ≤ θ ≤ 25.02°. A total of 12122 reflections were collected, of which 2669 were independent (Rint = 0.127) and 2406 were observed with I > 2σ(I). The calculations were performed with SHELXS-97 program [25] and the empirical absorption corrections were applied to all intensity data. The non-hydrogen atoms were refined anisotropically. The hydrogen atoms were determined with theoretical calculations and refined isotropically. The final full-matrix least squares refinement gave R = 0.089 and wR = 0.251 (*w = 1/[s^2^(F_o_^2^) + (0.1322P)^2^ + 9.386P]* where *P = (F_o_^2^ + 2F_c_^2^)/3*, S = 1.06, (Δ/σ) max = 0.121, Δρ_max_ = 0.94 and Δρ_min_ = −0.60 e Å^−3^. CCDC No. 856797 contains the supplementary crystallographic data for this paper. These data can be obtained free of charge via www.ccdc.cam.ac.uk/conts/retrieving.html (or from the CCDC, 12 Union Road, Cambridge CB2 1EZ, UK; Fax: +44 1223 336033; Email: deposit@ccdc.cam.ac.uk).

### 3.4. Antifungal Activity Assays

The *in vitro* antifungal activities of **4a**–**v** against *Colletotrichum orbiculare*, *Botrytis cinerea* and *Rhizoctonia solani* were evaluated using the mycelium growth rate test [26]. The method for testing the primary biological activity was performed in an isolated culture. Under sterile conditions, sample (1 mL) was added to the culture plates, followed by the addition of culture medium (9 mL). The final mass concentration was 50 μg/mL. Circle mycelium with a diameter of 4 mm was cut using a drill. The culture plates were cultivated at 24 ± 1 °C. The extended diameters of the circle mycelium were measured after 72 h. Propiconazole, a commercial fungicide, was used as a control, and sterile water was used as a blank. Three replications were performed. The relative inhibition rate of the circle mycelium compared to blank assay was calculated via the following equation:

Relative inhibition rate (%) = [(dex − dex')/dex] × 100%

where dex is the extended diameter of the circle mycelium during the blank assay; and dex' is the extended diameter of the circle mycelium during testing.

## 4. Conclusions

In summary, 22 new 3-[(5-aryl-1,3,4-oxadiazol-2-yl)methyl]benzo[*d*]thiazol-2(3*H*)-ones were designed and synthesized by reaction of (5-substituted-2-oxobenzothiazolin-3-yl)-acetohydrazide with various aromatic acids in POCl_3_ under reflux conditions. The structures of the title compounds possess both benzothiazole and 1,3,4-oxadiazole skeletons, and their structures were confirmed by ^1^H-NMR, ^13^C-NMR, IR, MS and elemental analysis. Furthermore, the structure of compound **4i** was determined by single-crystal X-ray diffraction. The biological evaluation showed that some of them exhibited moderate inhibition activity against *Colletotrichum orbiculare*, *Botrytis cinerea* and *Rhizoctonia solani*, and could be useful lead compounds for fungicide development.

## Supplementary Materials

Supplementary materials can be accessed at: http://www.mdpi.com/1420-3049/17/1/989/s1.
