# Supplementary material for: Synthesis, Structure and Antifungal Activity of New 3-[(5-Aryl-1,3,4-oxadiazol-2-yl)methyl]benzo[d]thiazol-2(3H)-ones
Source: Molecules. 2012 Jan 18;17(1):989–1001. doi: 10.3390/molecules17010989 (PMC6268515; doi:10.3390/molecules17010989)

**Supplementary**

**^13^C-NMR spectra of 4a ~ v**

^13^C-NMR spectra of 4a: C_16_H_11_N_3_O_2_S


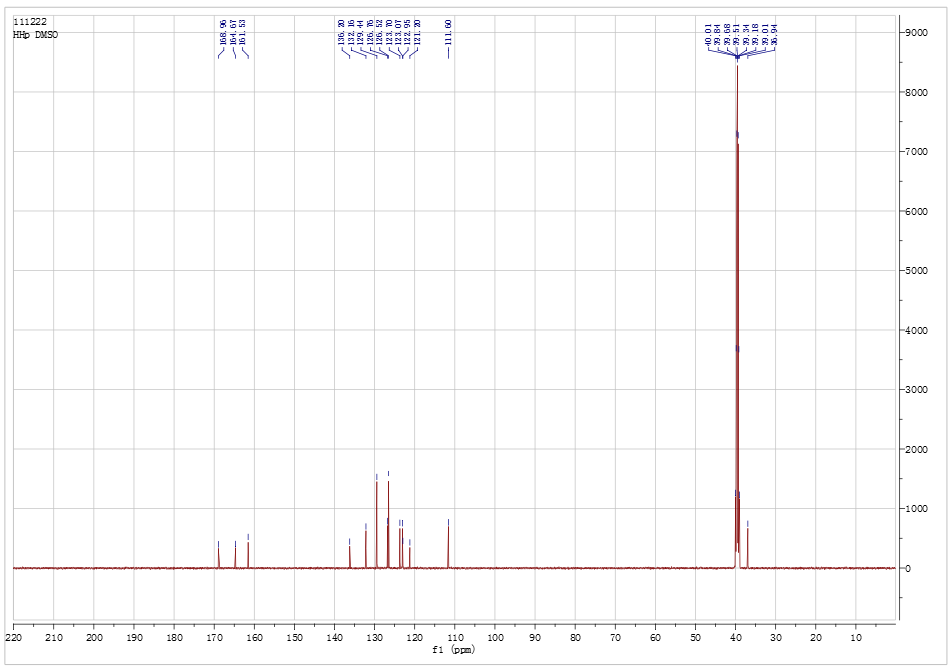


^13^C-NMR spectra of 4b: C_17_H_13_N_3_O_2_S


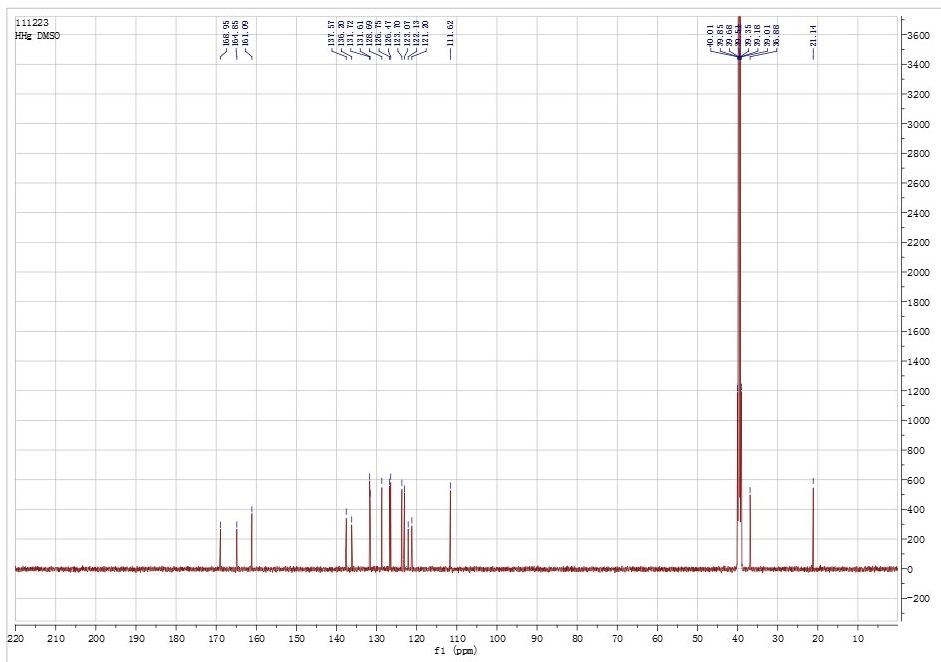


^13^C-NMR spectra of 4c: C_17_H_13_N_3_O_2_S


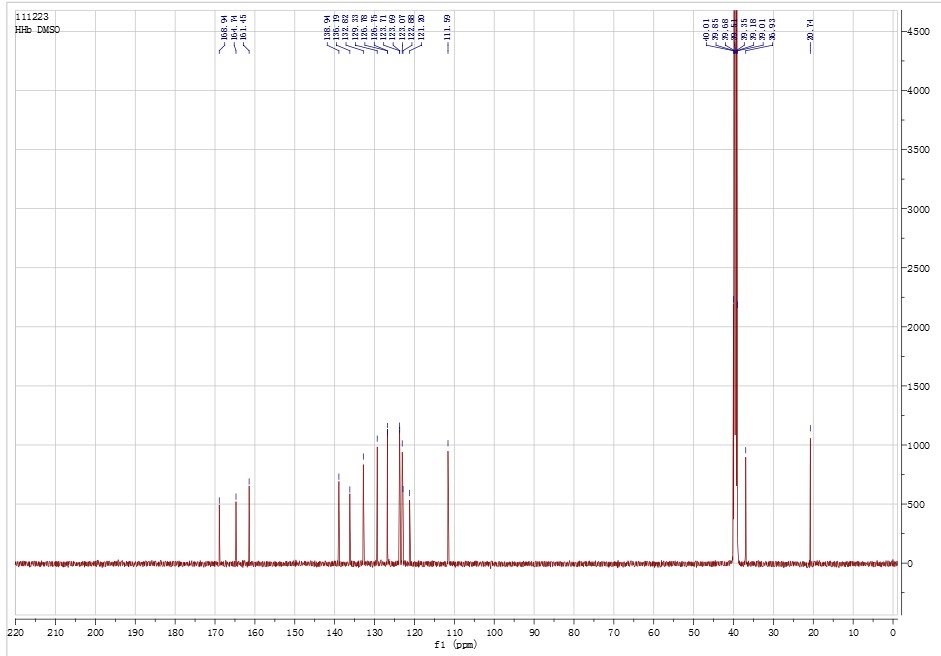


^13^C-NMR spectra of 4d: C_17_H_13_N_3_O_2_S


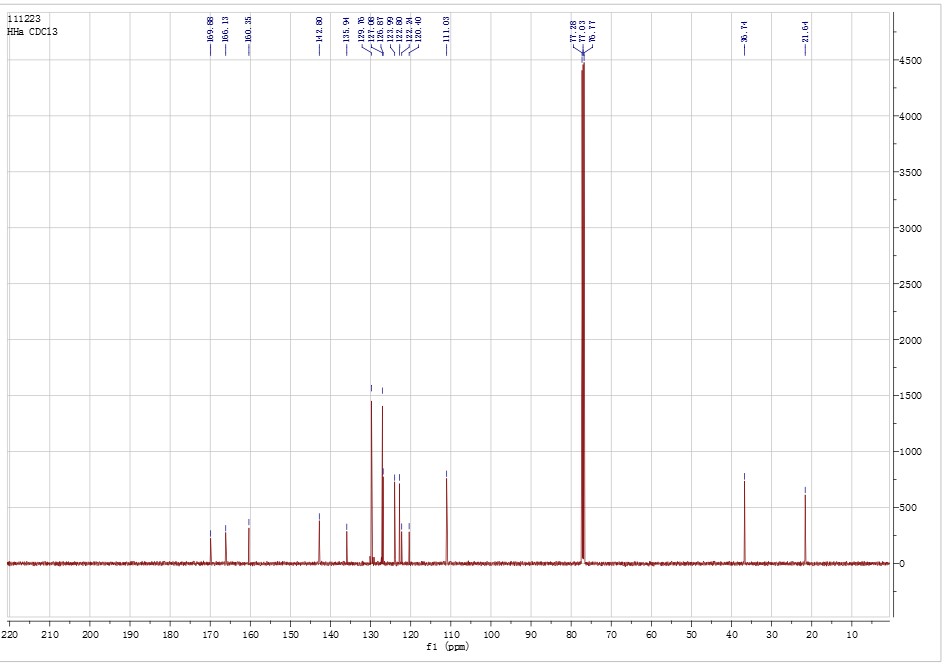


^13^C-NMR spectra of 4e: C_19_H_17_N_3_O_2_S


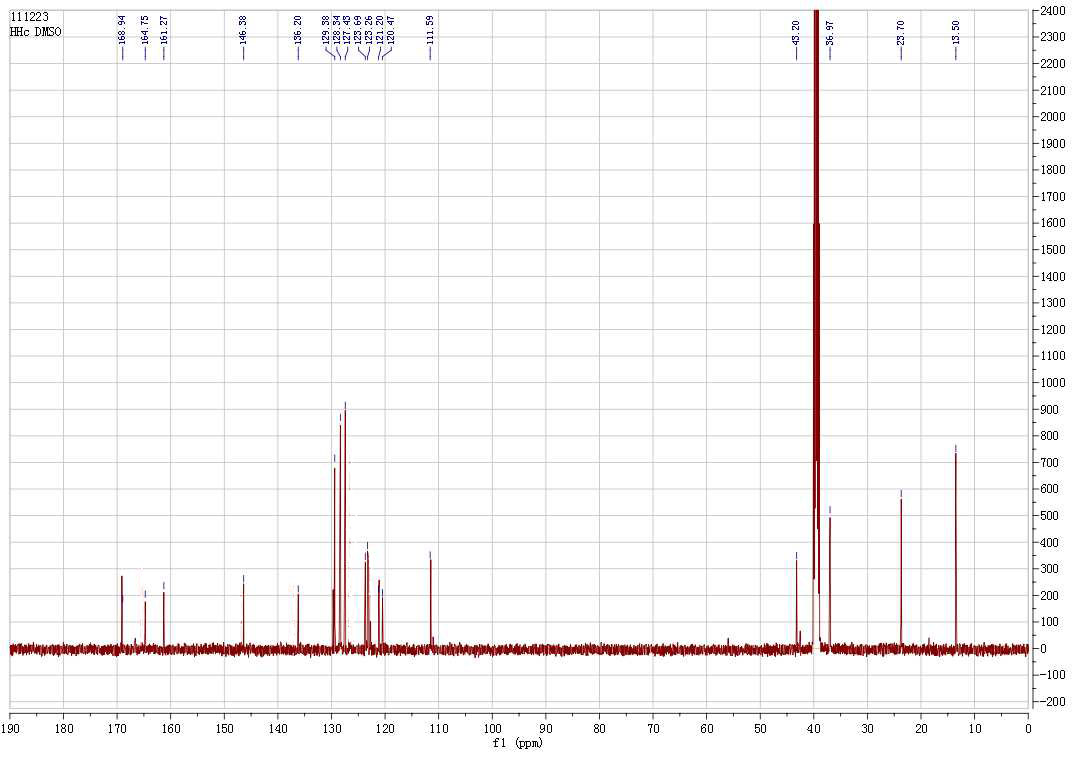


^13^C-NMR spectra of 4f: C_19_H_17_N_3_O_2_S


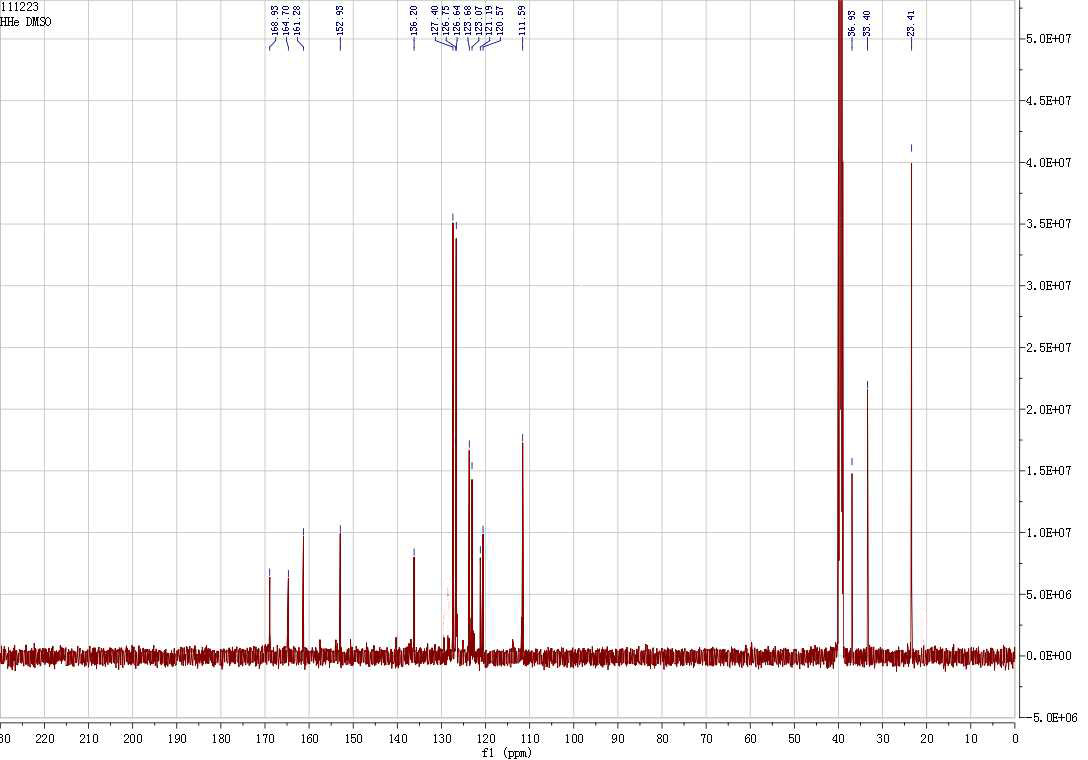


^13^C-NMR spectra of 4g: C_20_H_19_N_3_O_2_S


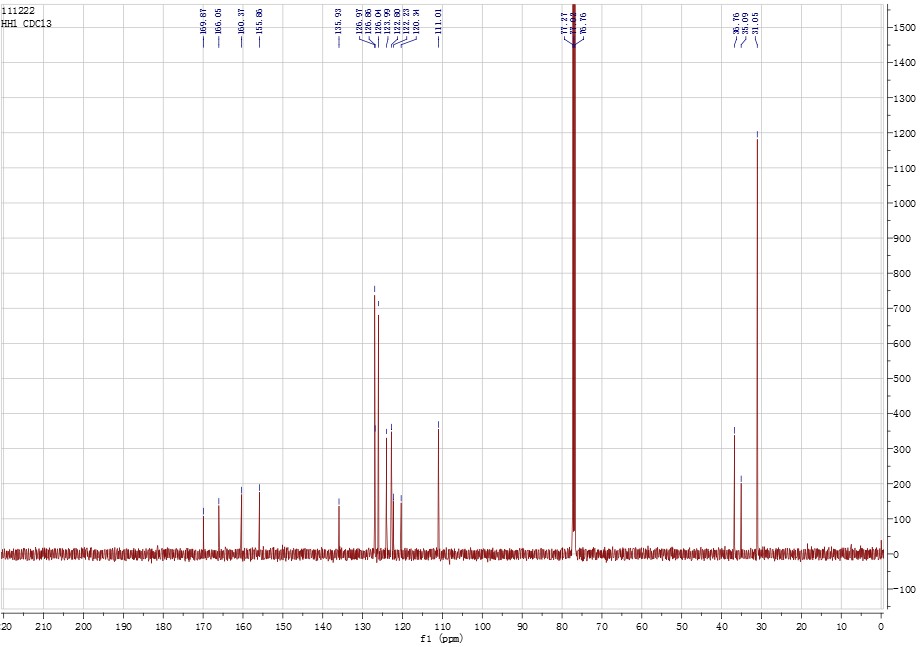


^13^C-NMR spectra of 4h: C_21_H_21_N_3_O_2_S


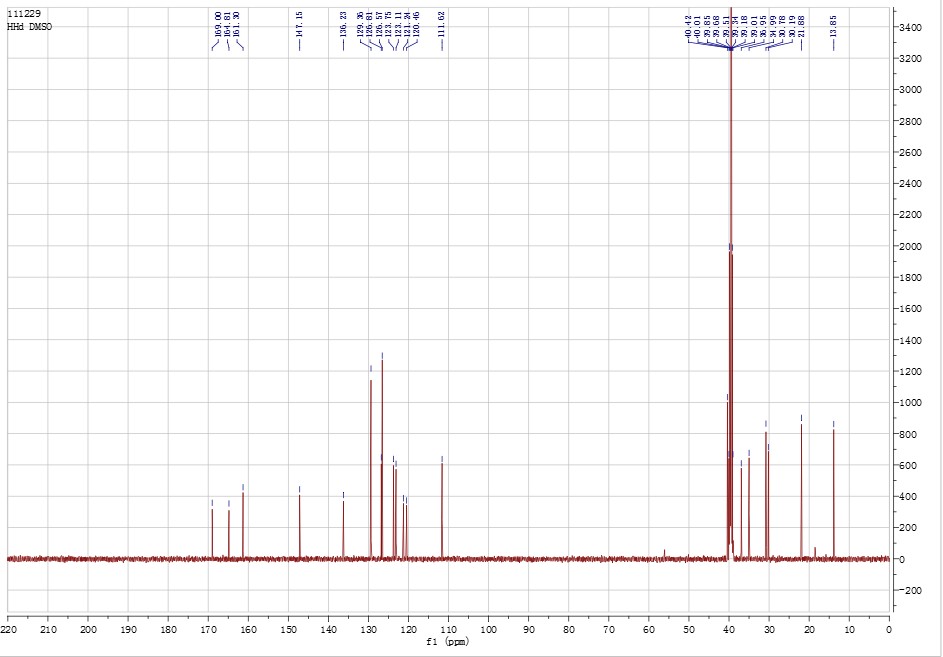


^13^C-NMR spectra of 4i: C_16_H_10_ClN_3_O_2_S


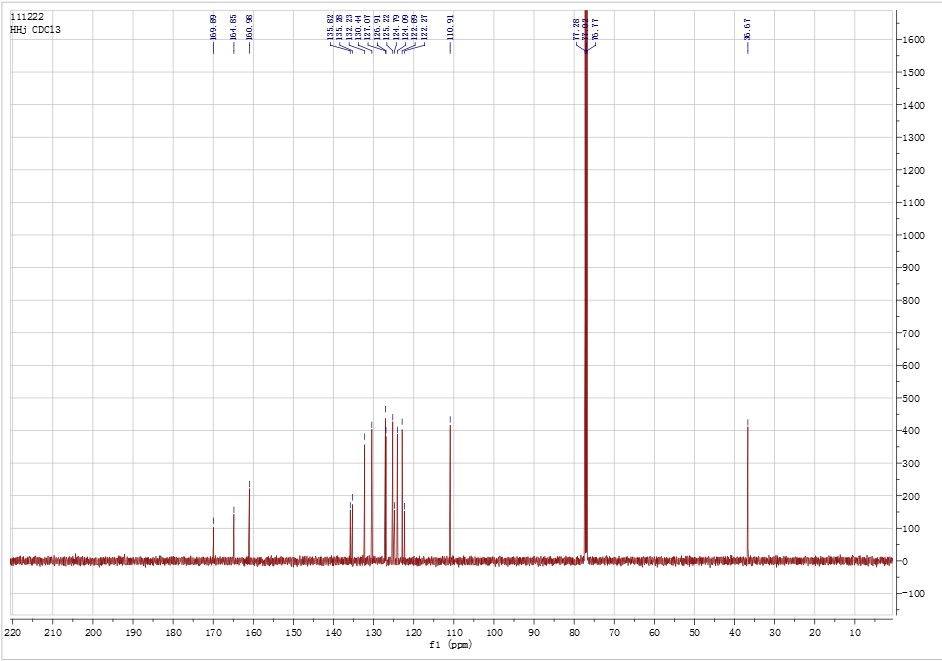


^13^C-NMR spectra of 4j: C_16_H_9_Cl_2_N_3_O_2_S


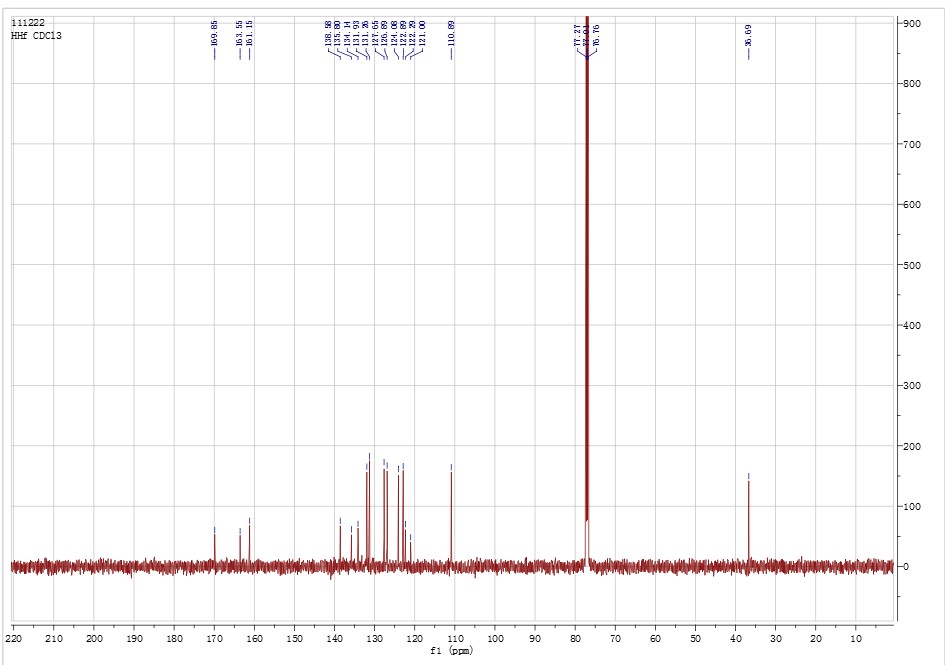


^13^C-NMR spectra of 4k: C_16_H_10_FN_3_O_2_S


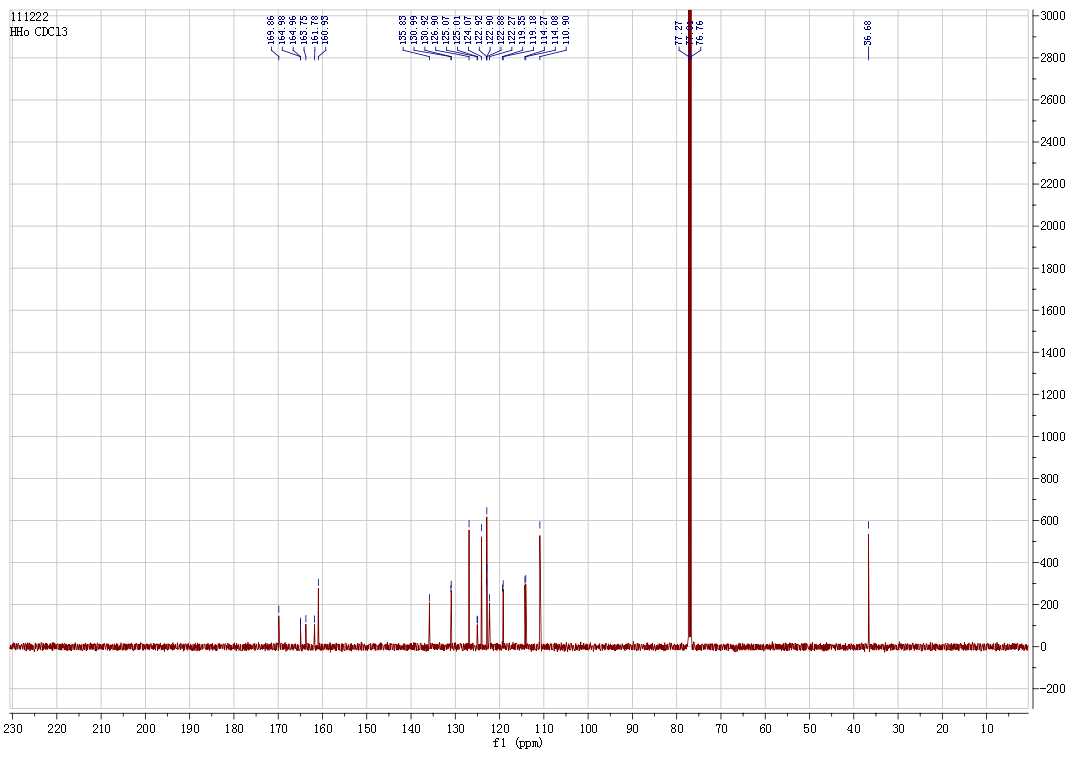


^13^C-NMR spectra of 4l: C_16_H_10_FN_3_O_2_S


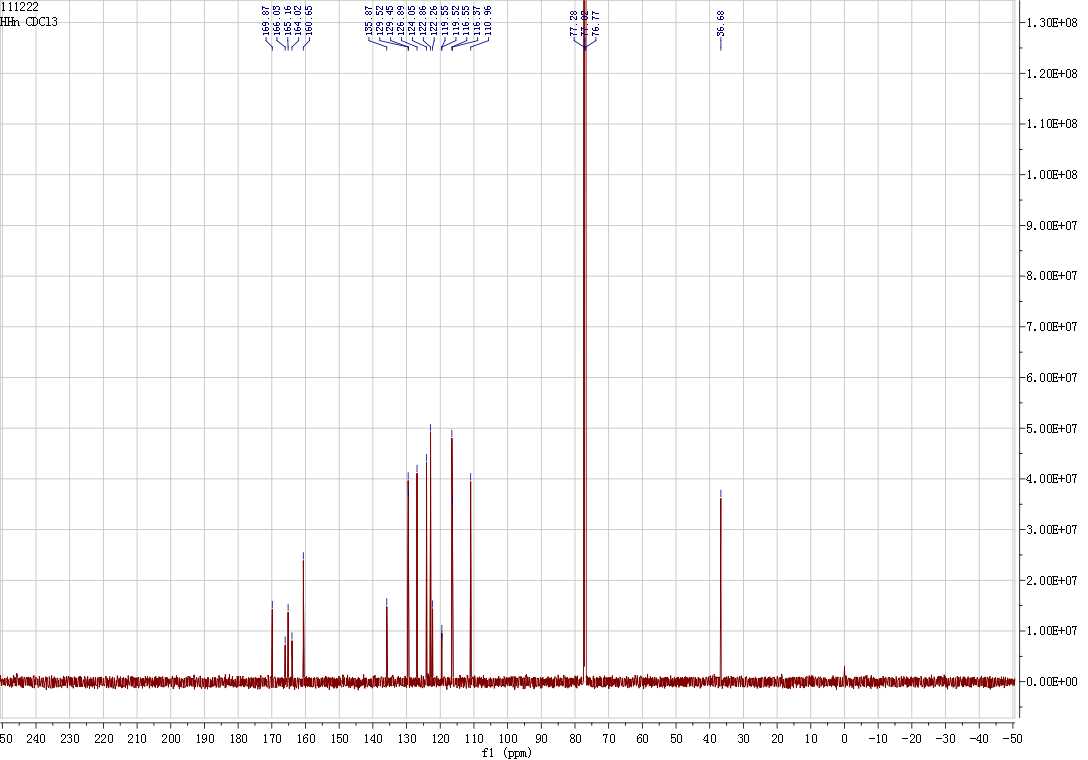


^13^C-NMR spectra of 4m: C_17_H_13_N_3_O_3_S


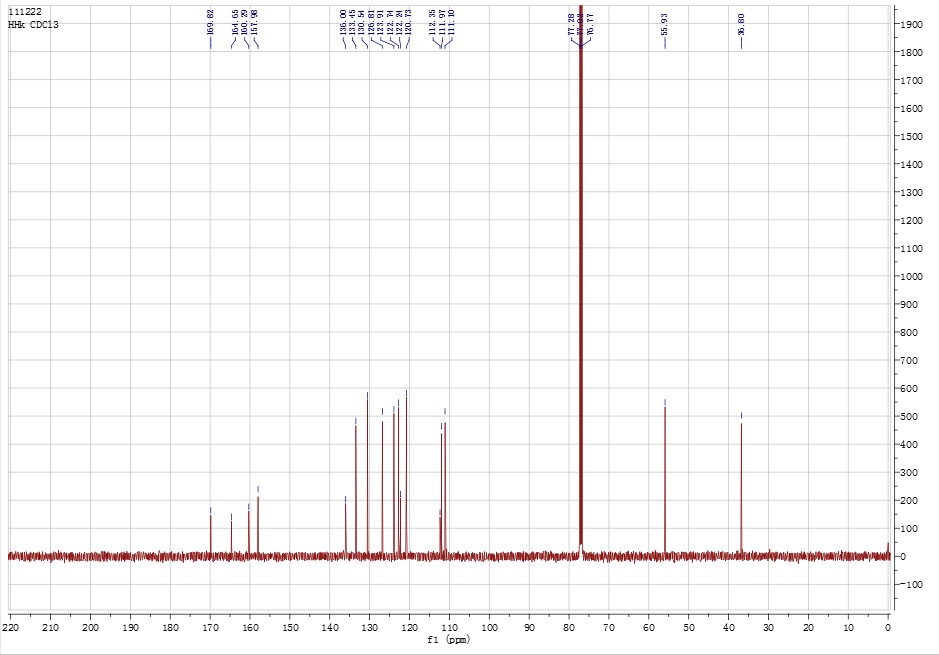


^13^C-NMR spectra of 4n: C_17_H_13_N_3_O_3_S


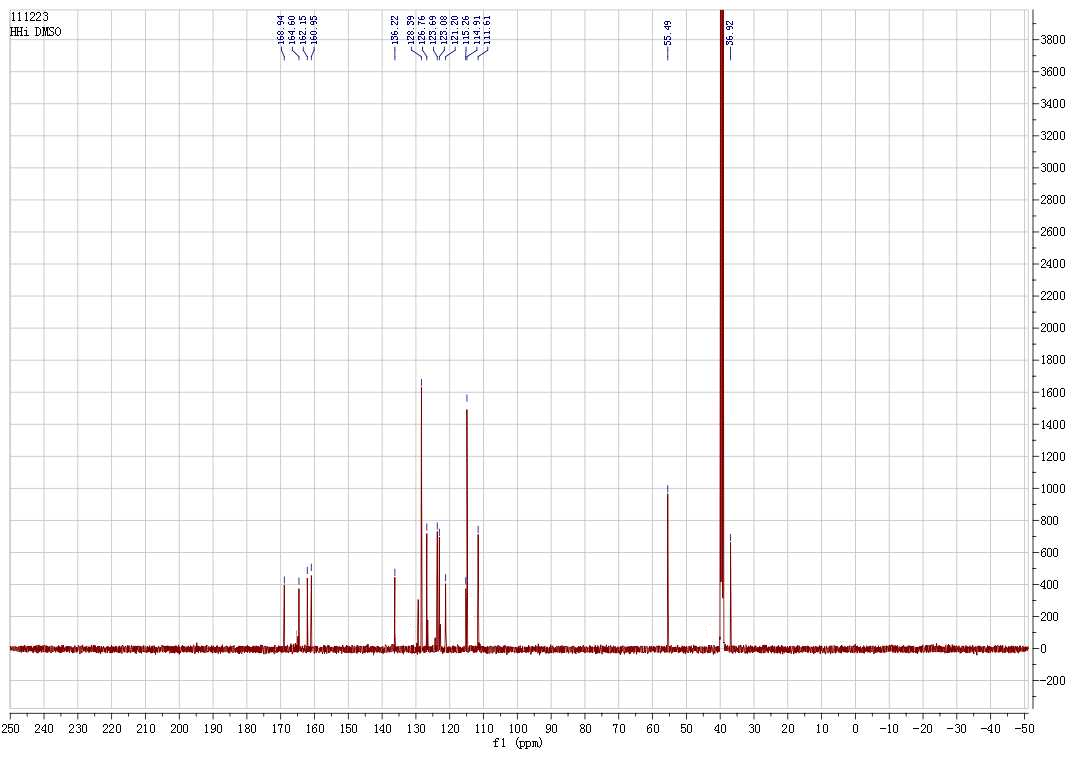


^13^C-NMR spectra of 4o: C_16_H_10_N_4_O_4_S


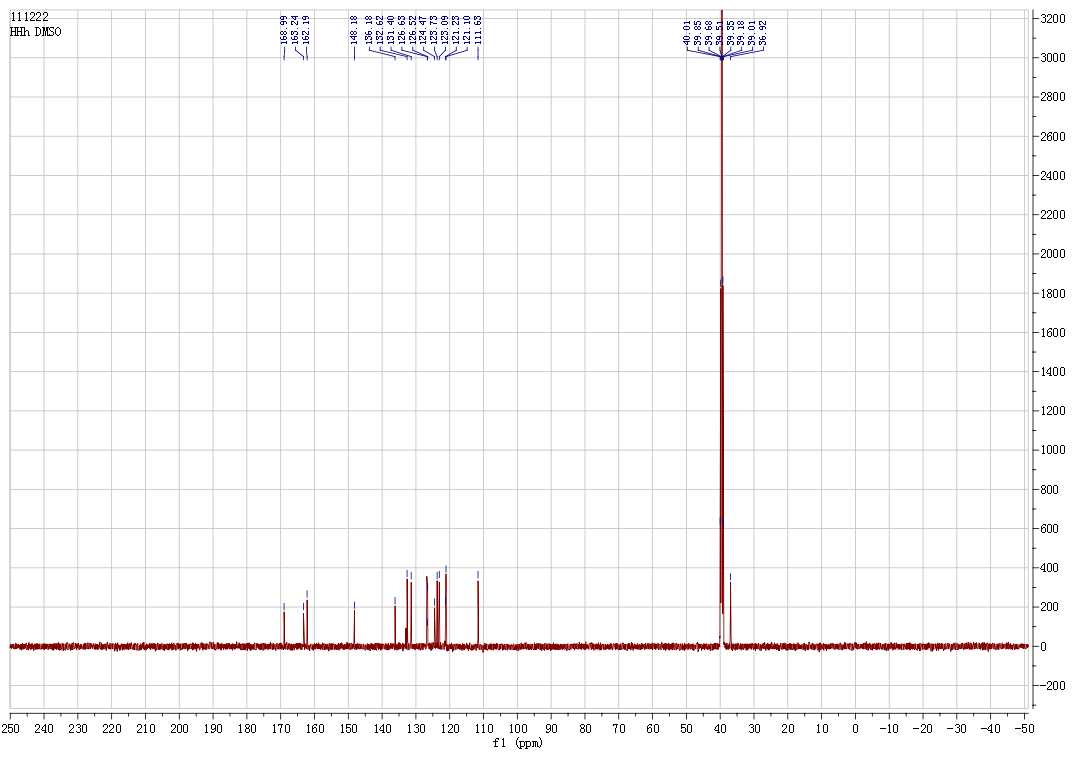


^13^C-NMR spectra of 4p: C_15_H_9_ClN_4_O_2_S


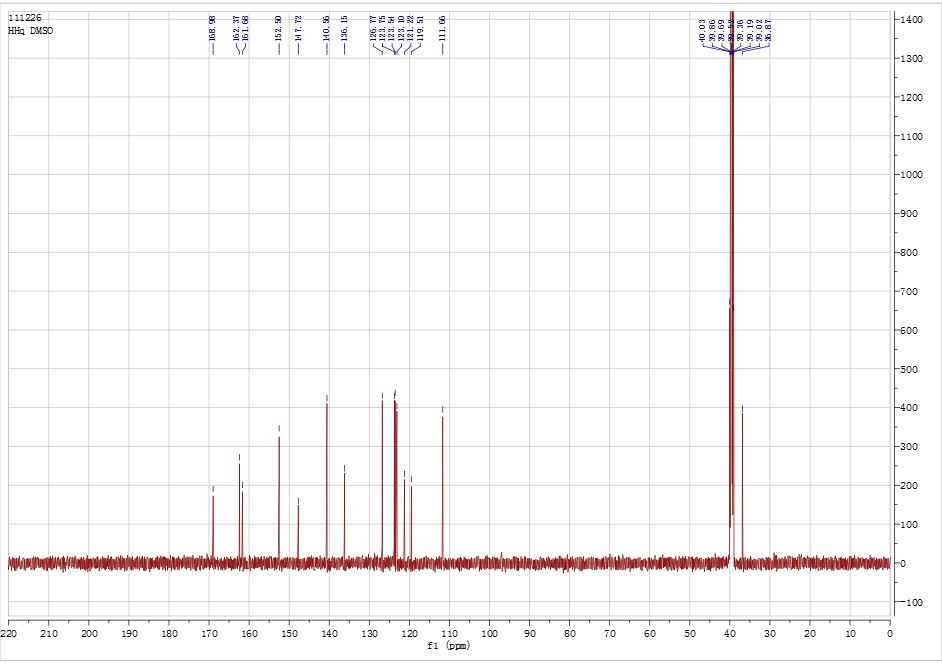


^13^C-NMR spectra of 4q: C_16_H_10_ClN_3_O_2_S


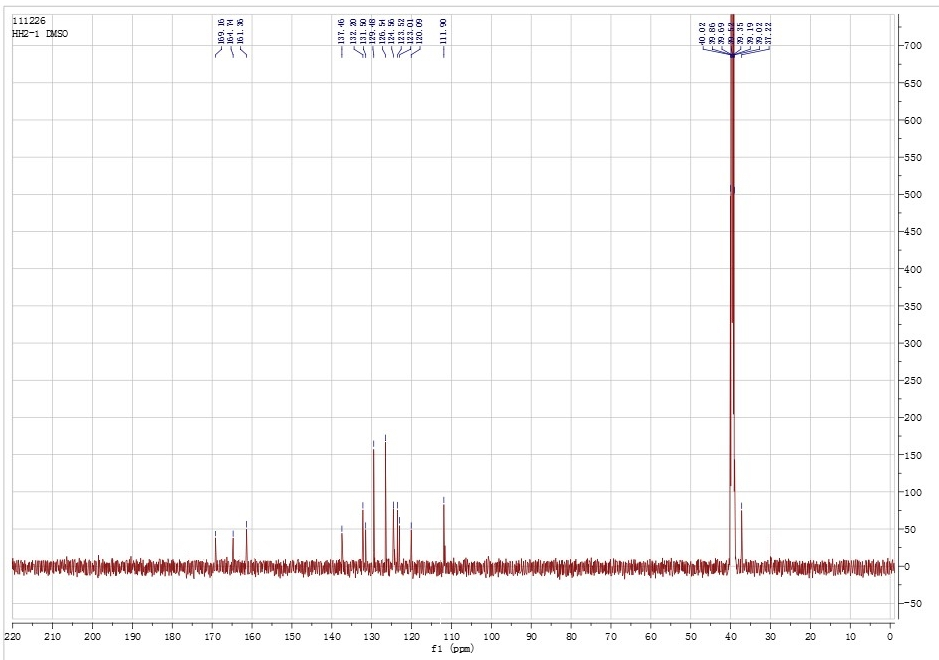


^13^C-NMR spectra of 4r: C_17_H_12_ClN_3_O_2_S


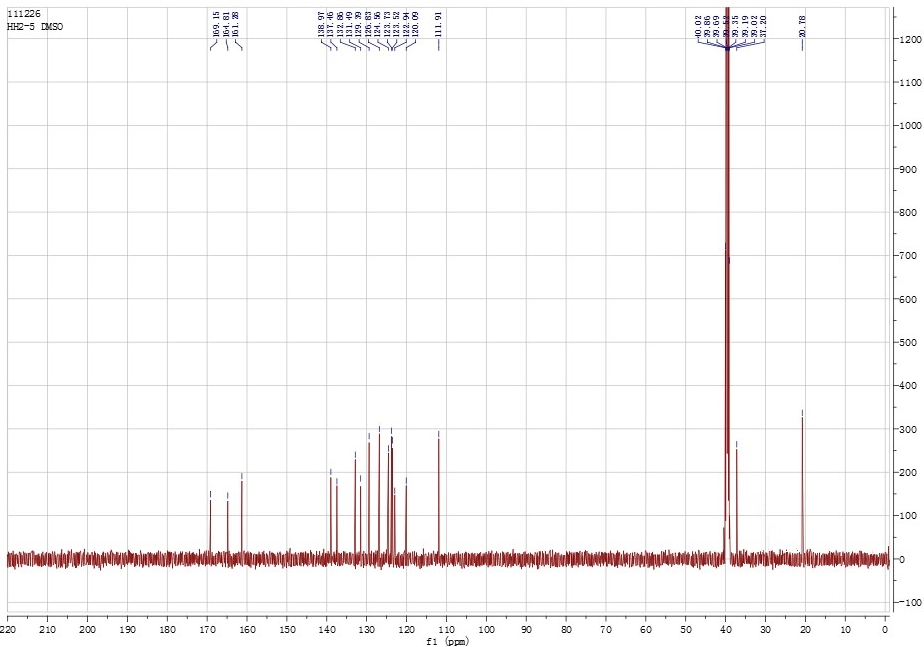


^13^C-NMR spectra of 4s: C_17_H_12_ClN_3_O_2_S


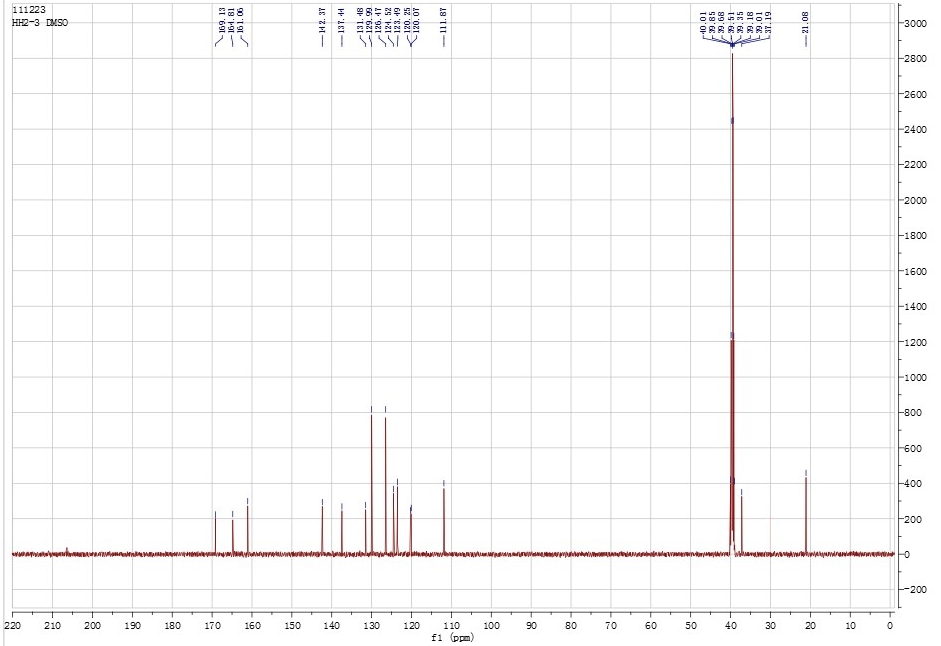


^13^C-NMR spectra of 4t: C_20_H_18_ClN_3_O_2_S


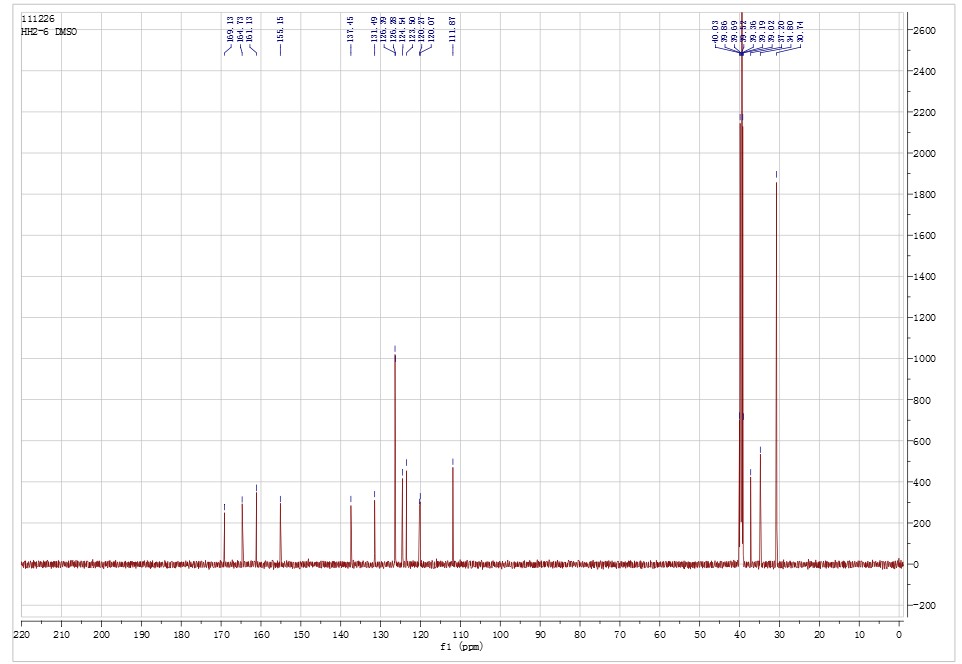


^13^C-NMR spectra of 4u: C_17_H_12_ClN_3_O_3_S


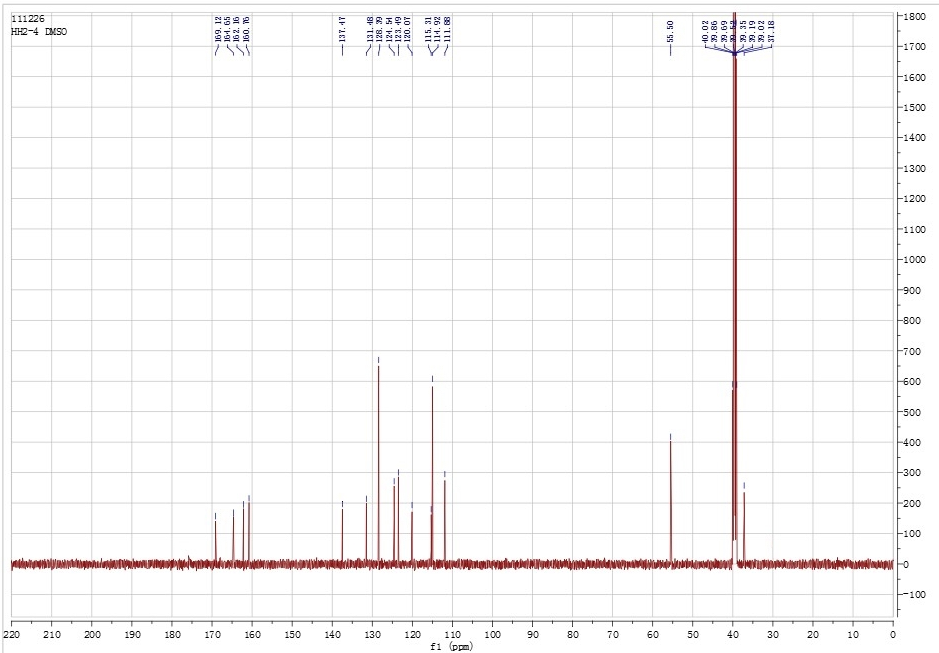


^13^C-NMR spectra of 4v: C_15_H_8_Cl_2_N_4_O_2_S


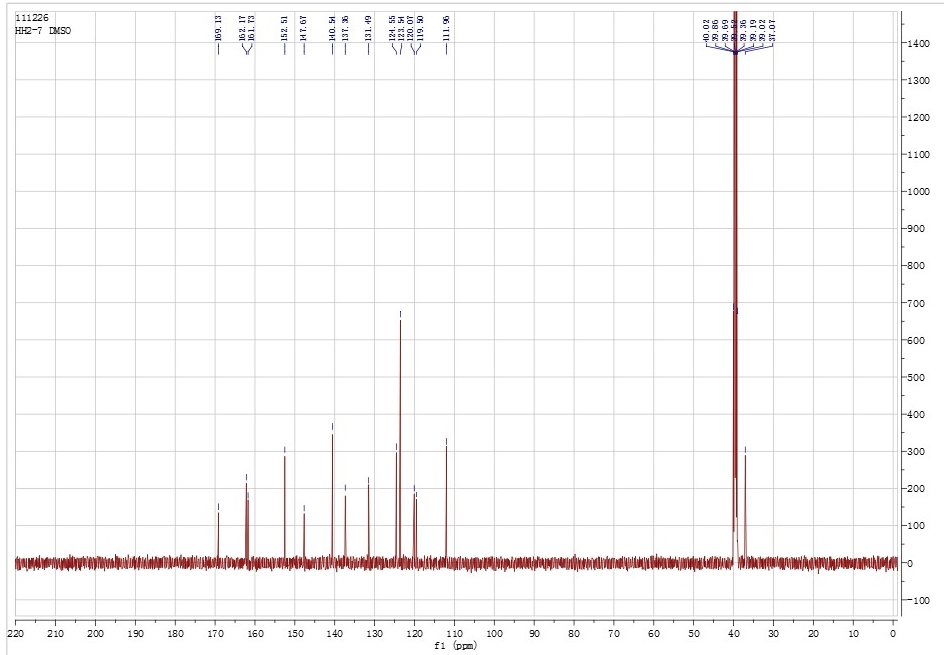

Supplement: Supplementary file 1 [file molecules-17-00989-s001.doc]
